# Risk Factors for Nipah Virus Encephalitis in Bangladesh^1^

**DOI:** 10.3201/eid1410.060507

**Published:** 2008-10

**Authors:** Joel M. Montgomery, Mohamed J. Hossain, E. Gurley, D.S. Carroll, A. Croisier, E. Bertherat, N. Asgari, P. Formenty, N. Keeler, J. Comer, M.R. Bell, K. Akram, A.R. Molla, K. Zaman, Mohamed R. Islam, K. Wagoner, J.N. Mills, P.E. Rollin, T.G. Ksiazek, R.F. Breiman

**Affiliations:** Centers for Disease Control and Prevention, Atlanta, Georgia, USA (J.M. Montgomery, D.S. Carroll, N. Keeler, J. Comer, M.R. Bell, K. Wagoner, J.N. Mills, P.E. Rollin, T.G. Ksiazek); International Centre for Diarrheal Diseases Research, Dhaka, Bangladesh (M.J. Hossain, E. Gurley, R.F. Breiman); World Health Organization, Geneva, Switzerland (A. Croisier, E. Bertherat, N. Asgari, P. Formenty); World Health Organization, Dhaka (K. Akram, K. Zaman); Institute of Epidemiology Disease Control and Research, Dhaka (A.R. Molla, M.R. Islam); 2 Current affiliation: US Naval Medical Research Center Detachment, Lima, Peru.

**Keywords:** Nipah virus, Bangladesh, encephalitis, paramyxovirus, bat, research

## Abstract

Nipah virus (NiV) is a paramyxovirus that causes severe encephalitis in humans. During January 2004, twelve patients with NiV encephalitis (NiVE) were identified in west-central Bangladesh. A case–control study was conducted to identify factors associated with NiV infection. NiVE patients from the outbreak were enrolled in a matched case-control study. Exact odds ratios (ORs) and 95% confidence intervals (CIs) were calculated by using a matched analysis. Climbing trees (83% of cases vs. 51% of controls, OR 8.2, 95% CI 1.25–∞) and contact with another NiVE patient (67% of cases vs. 9% of controls, OR 21.4, 95% CI 2.78–966.1) were associated with infection. We did not identify an increased risk for NiV infection among persons who had contact with a potential intermediate host. Although we cannot rule out person-to-person transmission, case-patients were likely infected from contact with fruit bats or their secretions.

## Abstract 2

Patients in Goalando were likely infected by direct contact with fruit bats or their secretions, rather than through contact with an intermediate host.

Henipaviruses (family *Paromyxoviridae*, genus *Henipavirus*) are enveloped RNA viruses that cause respiratory illness in pigs and horses and respiratory illness and encephalitis in humans (*1*–*6*). After a 4- to 18-day incubation period, human disease can rapidly progress from mild illness (fever, headache, myalgia) to coma and death within 10 days; the case-fatality ratio is 40%–76% (*3*,*7*–*10*). The first recognized human Henipavirus infections occurred in 1994 in Australia, where a respiratory disease among horses was associated with illness in 2 humans (*11*). The etiologic agent, Hendra virus, was subsequently isolated from asymptomatic flying foxes (fruit bats of the family *Pteropodidae*) (*12*). Field et al. (*2*) suggested that horses, identified as the intermediate hosts linked to human illness, may have become infected through indirect contact with fruit bats (e.g., infected fetal bat tissues or fluids).

The first reported human epidemic of encephalitis caused by another Henipavirus, Nipah virus (NiV), occurred between September 1998 and April 1999 in Malaysia and Singapore and was associated with an outbreak of severe respiratory illness in pigs (*13*–*15*). Most (86%–93%) human NiV encephalitis (NiVE) infections during this outbreak involved occupational exposure to pigs, implicating these animals as an intermediate host for NiV (*15*–*18*). Outbreaks of NiVE occurred in Bangladesh during 2001 and 2003, in areas where NiV antibody–positive fruit bats have been identified (*19*). These reports, in addition to ecologic surveys conducted in Cambodia, have strengthened evidence that pteropid bats are the reservoir for Hendra and Nipah viruses (*12*,*20*–*25*).

An outbreak of encephalitis in Bangladesh was recognized on January 21, 2004; it affected 2 villages of Goalando township, Rajbari District, Dhaka Division, 70 km west of the city of Dhaka (Figure 1). Ten deaths were reported among 12 ill persons with symptoms compatible with NiVE, resulting in a case-fatality ratio of 83% (*9*,*23*). Although previous outbreaks of NiVE outside Bangladesh involved primarily men and women >25 years of age (*5*,*16*,*17*,*19*,*26*), most (75%) patients in this outbreak were boys <15 years of age. We describe a matched case-control study that was conducted to characterize the epidemiology of NiVE and, specifically, to determine if risk for NiVE was associated with contact with animals; an environmental exposure, activity, or behavior; or contact with other NiVE patients during the 2004 NiVE outbreak in Goalando township.

**Figure 1:**
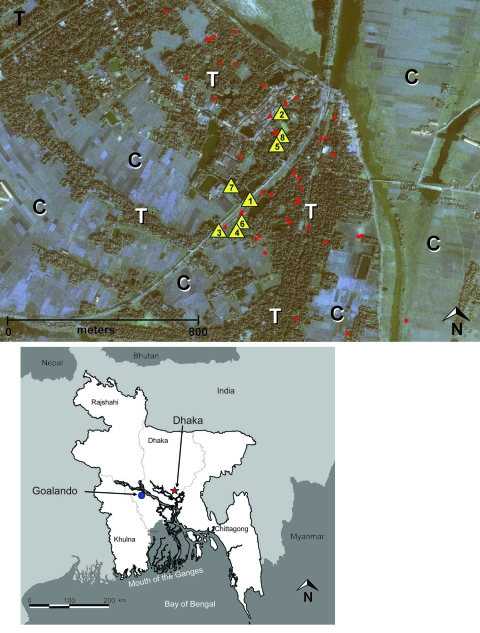
Top: Distribution of Nipah virus case (n = 12) and control (n = 36) households within the outbreak/study site of Goalando township, Bangladesh, January 2004. Number in the yellow triangle corresponds to household no. in Figure 2. Map also shows extreme habitat disturbance; areas under cultivation (for rice, sugar cane) are highlighted with “C,” and remaining trees (fruit trees and bamboo stands) with “T.” Bottom: Location of outbreak village.

## Materials and Methods

### Study Participants

A matched case-control study was conducted in Goalando, Bangladesh (Figure 1), February 18–22, 2004. Hypotheses tested in this study, as mentioned above (e.g., increased risk for NiV infection caused by contact with animals, environmental exposure, contact with fruit in season) were based upon factors associated with previous outbreaks of NiVE in Malaysia, Singapore, and Bangladesh.

### Case Definition

A confirmed NiVE case-patient was defined as any patient with fever and symptoms compatible with encephalitis after December 15, 2003, with NiV-specific immunoglobulin M antibodies in cerebrospinal fluid (CSF) or serum by enzyme immunoassay (EIA). A probable case of NiVE was defined as a patient with a diagnosis of encephalitis in whom fever developed and who was living in the same village as a patient with a confirmed case of NiVE after December 15, 2003. Cases remained in the probable category if the patient died and a specimen for laboratory confirmation could not be obtained.

We conducted a population census of the affected area in February 2004; this census was the basis for selecting controls. We identified 3 controls for each case-patient. The controls were selected randomly from the population and then matched to each case-patient on the basis of gender and age group. All households identified during the census, including houses of case-patients and controls, were mapped by Global Positioning System, and data were uploaded into ERDAS Imagine 8.5 (Leica Geosystems, Atlanta, GA, USA) and merged with a November 2000 IKONOS Geo 1-m satellite image of the outbreak area (Space Imaging, Thornton, CO, USA).

Participation was strictly voluntary, and written informed consent was obtained for all participants; for those <18 years of age, individual and parental consent was obtained. The Bangladesh Ministry of Health and Family Welfare that requested this investigation reviewed and approved all protocols.

### Study Population

**Figure 2:**
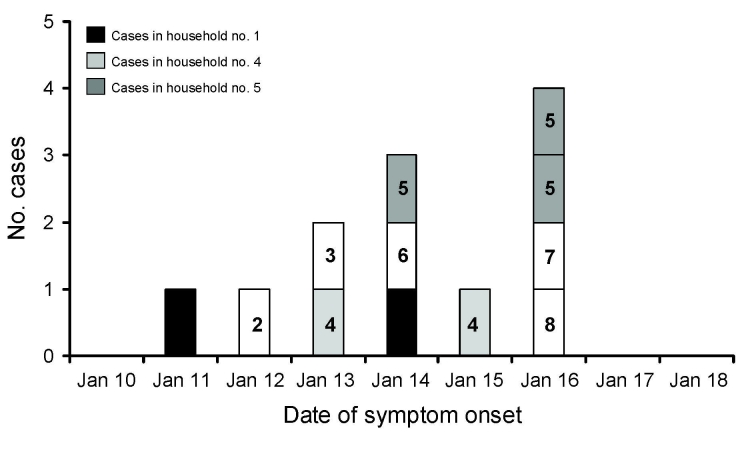
Epidemic curve of Nipah virus outbreak in Goalando, Bangladesh, in 2004, demonstrating household clustering. Households 1 and 4 each had 2 cases, household 5 had 3 cases, and all other households, single cases.

Probable and confirmed cases identified in 2 contiguous villages of Goalando township (Figure 1) were included in this study. Seven of the 12 cases were clustered within 3 households. Of these 7 clustered cases, 3 occurred in 1 household, and the remaining 4 were distributed in 2 separate homes (Figures 1, 2). Therefore, we conducted 2 separate analyses to assess the effect of case clustering on results. The first analysis contained the complete dataset of 12 cases and 36 controls; the subanalysis consisted of 8 cases (we randomly selected 1 case/household) and 24 matched controls. Similar results (proportions, odds ratios [ORs], 95% confidence intervals [CIs]) were obtained from both analyses. Thus, data presented in this article, including all tables, are derived from the complete dataset.

### Specimen Collection and Testing

Serum samples and CSF were tested as previously described (*27*). When possible, a serum specimen was collected from controls.

### Data Collection and Interviews

After informed consent was obtained, case-patients and controls were interviewed at home by trained interviewers, in their native Bengali language, with a standardized questionnaire. Information such as demographics, types of animal exposures, environmental and occupational exposures, exposure to ill persons, and history of illness was obtained. Proxy interviews of family members and/or friends were conducted for deceased patients. To minimize interview bias, proxy interview methods were also used for all controls that were matched to deceased case-patients.

### Statistical Analysis

Exact ORs and 95% CIs were calculated by using a matched univariate logistic regression analysis in SAS version 9.0 (SAS Institute Inc, Cary, NC, USA) (*28*). Associations were considered statistically significant at p<0.05.

## Results

### Descriptive Characteristics

Four (33%) cases were confirmed by EIA; the remaining 8 (67%) case-patients, from whom a diagnostic specimen was not available, were considered probable cases. Among all 13 (36%) controls who consented to blood collection, results of serologic tests for NiV-specific antibodies were negative. Furthermore, none of the controls reported having had a perceived fever or symptoms compatible with NiVE from December 15, 2003, through the week the study was conducted (February 18–22, 2004). In addition, an antibody prevalence study conducted among persons (n = 300) living in the outbreak site showed no evidence of asymptomatic or mild infection, which suggested that controls entered into the study were likely uninfected (A. Croisier, unpub. data). Proxy interviews were administered to equal proportions of case-patients (83%) and controls (Table 1). The median age of case-patients included in the study was 11.5 years (range 2–28 years); 9 (75%) were male, and 11 (91%) were <15 years of age (Table 1). Residences of all case-patients and controls were located within the affected villages, an area with a radius of ≈800 m (Figure 1).

**Table 1:** Descriptive characteristics of Nipah virus case-patients and controls, Bangladesh, January 2004

Characteristic	No. (%)	
	Case-patients, n = 12	Controls, n = 36
Sex		
M	9 (75)	27 (75)
F	3 (25)	9 (25)
Age group, y		
1–5	1 (8)	3 (8)
6–10	4 (33)	12 (33)
11–15	6 (50)	18 (50)
16–20	0	0
21–25	0	0
26–30	1 (8)	3 (8)
Interview type		
Proxy	10 (83)	30 (83)
Self	2 (17)	6 (17)

### Animal Exposures

In the matched case-control analysis, a greater percentage of case-patients (60%) than controls (34%) had observed or touched dead animals, although this finding was not statistically significant (Table 2). We observed no differences between case-patients and controls with respect to contact with ill animals (Table 2), including pigs, ruminants, and fruit bats. Chickens and ducks were often slaughtered for religious purposes or for consumption; however, close contact with these animals and their bodily fluids (e.g., blood, saliva) during this process was not associated with NiV infection (Table 2). None of the case-patients or controls had known contact with pigs (healthy or ill) or pig excreta (Table 2). Four (36%) of 11 case-patients and 7 (19%) of the controls observed fruit bats around their household during the night (OR 4.1, p = 0.49; Table 2). However, some proxy family members and/or friends answering on behalf of patients who had died were unable to answer specific questions (e.g., Did you observe fruit bats around your house during the night?).

**Table 2:** Exposures and activities associated with Nipah virus infection, Bangladesh, December 2003–January 2004*

Exposure or activity	No. (%) study participants with reported exposure or activity†			
	Case-patients, n = 12	Controls, n = 36	OR (95% CI)	p value‡
Animal exposure				
Touched any ill animal§	9 (75)	31 (85)	1.8 (0.29–8.52)	0.613
Touched or observed a dead animal§	6/10 (60)	12 (34)	2.4 (0.4–616.5)	0.392
Killed any animal§	3 (25)	6 (16)	1.8 (0.2–79.51)	0.670
Other animal exposures				
Contact with animal stool	2/9 (22)	12 (35)	0.5 (0.05–3.04)	0.679
Visited a poultry farm	3 (25)	13 (37)	0.6 (0.08–3.29)	0.740
Observed fruit bats around household at night (1 mo before outbreak)	4/11 (36)	7 (19)	4.1 (0.27–261.9)	0.491
Outdoor activity				
Climbed trees	10 (83)	19 (51)	8.2 (1.25–∞)	0.025
Picked fruit from trees	8 (67)	18 (49)	3.2 (0.54–36.0)	0.262
Picked fruit from the ground	7/11 (64)	27 (74)	0.79 (0.13–6.09)	1.000
Fished	6 (50)	10 (28)	4.5 (0.69–49.7)	0.139
Hunted	2/10 (20)	10 (28)	7.3 (0.38–432.6)	0.240
Played hide and seek	8/11 (73)	21 (58)	4.3 (0.38–∞)	0.256
Played cricket	4 (33)	18 (51)	0.5 (0.09–2.76)	0.552
Played soccer	5 (42)	9 (24)	2.4 (0.44–16.9)	0.403
Exposure to human illness				
Had contact with a suspect or probable Nipah virus encephalitis case-patient	8 (67)	3 (9)	21.4 (2.78–966.1)	<0.001
Visiting a hospital	12 (100)	7 (19)	32.4 (5.18–∞)	<0.0001
Consumption of fruit				
Bananas¶	11 (92)	24 (67)	4.9 (0.61–226.7)	0.199
*Buroys*	7 (58)	28 (77)	0.4 (0.078–2.37)	0.433
*P*apaya	3 (25)	14 (40)	0.49 (0.08–2.24)	0.497
*Gu*ava	2 (17)	12 (33)	0.5 (0.05–2.70)	0.608
*Sofeda*	1 (8)	2 (5)	2.0 (0.03–38.4)	0.976
*Kamranga*	1 (8)	3 (9)	1.0 (0.006–165.9)	1.000
Other environmental exposures				
Drinking raw DPS	10/11 (91)	26 (72)	4.1 (0.47–197.0)	0.328
Harvesting DPS	3 (25)	3 (8)	3.4 (0.37–43.6)	0.365
Drinking DPS from collection vessel	5/10 (50)	12 (32)	1.7 (0.36–8.34)	0.612
Someone in household collects DPS	4 (33)	5 (15)	2.3 (0.38–13.3)	0.454

*OR, odds ratio; CI, confidence interval; DPS, date palm sap. †Data are no. of study participants responding affirmatively/total no. responding (%) unless otherwise noted. ‡Exact method using univariate conditional logistic regression. §Cows, horses, sheep, goats, pigs, ducks, chickens, dogs, cats, or fruit bats. ¶Fruit was obtained from a market or another person, if not picked directly from the tree or ground.

### Environmental and Behavioral Exposures

A greater proportion of case-patients (83%) than controls (51%) reported having climbed trees between December 15, 2003, and February 3, 2004 (OR 8.2, p = 0.025; Table 2). No statistically significant differences were observed between case-patients and controls with respect to outdoor activities such as hunting, fishing, or playing outdoor games (e.g., hide-and-seek, cricket, soccer). Eating fruit that was locally available (on trees or collected from fruit trees locally) between December and February was not associated with illness, regardless of how the fruit was collected (from the ground, picked from tree, from the market) (Table 2). Although a greater proportion of case-patients reported environmental exposures (drinking raw date palm sap, harvesting date palm sap, having someone in the household who collects date palm sap, or drinking sap directly from the collection vessel), these differences were not statistically significant (Table 2).

### NiVE Case Exposure

There were strong associations between illness and 1) visiting a hospital and/or 2) having had contact with a probable or confirmed NiVE patient (Table 2). In one 2-case family cluster, a mother (26 years of age) and her infant son (2 years of age) both became ill and died. The child became symptomatic 2 days before the mother’s illness onset (Figure 2; household 4). Among the other affected family clusters, the patients became ill within 3 days of one another (Figure 2; households 1 and 5); all persons in these 2 clusters reported a history of climbing fruit trees. There was no evidence of contact of persons between case households during their illness.

## Discussion and Conclusions

In contrast to the patients in the Malaysian and Singapore outbreaks, which occurred primarily among adults, a preponderance of the NiV patients in the January/February 2004 Bangladesh outbreak were young boys. These findings, in the absence of high infection rates among adults or evidence of antibodies to NiV in the general population (investigation team, unpub. data), suggest an association between NiV infection and some childhood activity or specific behavior. The odds of NiV infection were significantly elevated among persons who climbed trees, an activity observed almost exclusively among boys <15 years of age. This behavior is quite common among children because they gather fruit from trees. Therefore, these children may have had contact with partially eaten fruit from fruit bats or the secretions/excretions of these animals. Or, the children may have contacted contaminated fruit bat guano or urine in the trees. The percentages of case-patients playing hide-and-seek, hunting, and fishing—all of which were typical behaviorial traits of local boys—were not significantly different than those for controls. These activities generally occur outdoors; however, they do not place a child in direct contact with bat excretions or secretions, as may be true for tree climbing. Therefore, infection was apparently related to a specific behavior, tree climbing, rather than age or outdoor activities in general. Furthermore, although other exposures that may have placed persons in closer contact with bat secretions (e.g., collecting fruit or palm sap from trees, drinking palm sap directly from collection vessel) were observed more often among case-patients than controls, these findings were not statistically significant; perhaps because of the small sample size. Nonetheless, our findings can and have been used to help guide NiV outbreak investigations, leading investigators to similar conclusions as ours (*29*).

Fruit bats forage at night in various trees that are producing ripe fruit and often drink from palm sap collection vessels (*30*). Fruits are also a major food source for many villagers and, as a result of environmental disturbances (*31*) in the form of crop development (e.g., jute, rice, and sugar cane), the few remaining fruit trees grow only in close proximity to human dwellings (Figure 1). This in turn creates a situation in which fruit bats are forced into close proximity with humans, especially while these mammals are foraging and feeding. In addition, date palm sap is routinely collected in rural areas of Bangladesh between December and May. According to villagers, including palm sap harvesters, dead fruit bats are occasionally found in the collection vessels. Local villagers reported that they often observed fruit bats feeding from palm sap collection vessels, and some collectors place cloth over the opening of the vessel to prevent this (investigational team observation). In fact, a greater proportion of case-patients in our study collected palm sap, drank from the palm sap collection vessel, or had a family member who collected palm sap; however, these differences were not statistically significant. The power of our study to detect exposure risks was limited by the outbreak size. Therefore, until additional data are available, remaining cautious of date palm sap collection vessels, especially those visibly contaminated with fruit bat excreta or carcasses, would be prudent.

Numerous investigators have found serologic evidence suggesting that fruit bats of the genus *Pteropus* are the reservoir hosts for NiV (*23*,*24*), and there are reports of NiV isolation from bat urine (*20*,*25*) and partially eaten fruit (*20*). Unpublished laboratory data from the Bangladesh investigation have not supported the presence of an intermediate or primary reservoir host other than *P. giganteus*. Available data from this study, therefore, suggest direct transmission of NiV to humans through contact with bat secretions or excretions (saliva, urine, guano, partially eaten fruit) during fruit-tree climbing.

Although indirect contact with bats may have been the primary means of infection for this outbreak, Hsu and others (*19*) demonstrated that contact with ill cows was associated with an increased risk for NiV infection during the 2001 Bangladesh NiV outbreak. Therefore, intermediated hosts should be considered in future NiV outbreaks in Bangladesh.

In contrast to the patients in the Malaysia and Singapore outbreaks (*5*,*16*,*17*,*25*,*26*), most of the Bangladesh population (and all of the case-patients included in this study; data not shown) are practicing Muslims who do not consume pork and who avoid contact with pigs. None of the case-patients and controls in our study population reported any contact with pigs or pig excreta, so it is unlikely that these animals played a role in this outbreak.

Clustering of cases within households was a prominent feature of this outbreak (Figure 2); 1 household contained 3 case-patients, all brothers of ages 7–15 years. However, the longest estimated incubation periods (duration from symptom onset to first known exposure to a NiVE family member) within the clusters reported here were less than the currently recognized 4-day minimum (*7*). This finding suggests that the family clustering may have resulted from a common source of infection (e.g., a specific tree they climbed, fruit they consumed, or palm sap collection vessel they were in contact with) rather than person-to-person transmission. Our data also show strong associations between NiV infection and visiting a hospital. However, because the participants were asked if they had visited a hospital within a range of dates (December 15, 2003–February 3, 2004) and not a specific date, we were unable to determine if they were ill with NiV before visiting the hospital or whether they acquired their infection there. Some accounts in the literature suggest person-to-person transmission of NiV; therefore, it is plausible that someone could acquire, through contact with a patient’s secretions or excretions, an NiV infection while visiting a hospital (*6*,*10*,*20*). Nevertheless, the most probable explanation for the observed association is that NiV encephalitis patients during this outbreak were severely ill, requiring hospitalization.

Although person-to-person transmission may have occurred in this outbreak, the initial infection (index case) may have occurred through contact with bat secretions rather than contact with an intermediate host. A limitation of our study is that we were unable to identify a specific mechanism by which person-to-person transmission may have occurred. NiV has been isolated from the respiratory secretions and urine of patients in the Malaysia, Singapore, and current Bangladesh outbreaks (*3*,*8*,*32*,*33*), which suggests a potential for NiV to be transmitted from person to person. Data based upon chain-of-transmission events and clustering of cases during other 2003 and May 2004 Bangladesh outbreaks led investigators to conclude that human-to-human transmission may have occurred (*3*,*19*). Therefore, given the potential for household or nosocomial transmission, we recommend the use of personal protective equipment (i.e., gloves, masks, gowns, and eye protection); strict hand hygiene and surface disinfection during and after contact with an NiVE patient; isolation of patients with confirmed or suspected NiV infection; and proper disposal of potentially contaminated materials.

In summary, tree climbing, a behavior largely engaged in by young boys, was associated with an increased risk for NiV infection; although the exact mode of transmission is unclear. Our data do not rule out the potential for person-to-person transmission. If person-to-person transmission were extremely efficient, the conditions and population density of Bangladesh (≈1,000 persons/km^2^; total population 141 million/144,000/km^2^) may have resulted in a much larger outbreak. Indeed, a study among health workers in Bangladesh did not find evidence of incidental transmission to persons caring for patients hospitalized with Nipah-related illnesses (*34*). Bat-to-human was the most probable route of transmission in Goalando; however, some undetermined intermediate or incidental hosts cannot be ruled out. Periodic introductions of NiV to human populations in this region may continue to occur because of the overlapping nature of human and pteropid bat habitats. Moreover, bat–human interactions are likely to increase due to bat habitat loss because the few fruit trees that remain will likely be found in close proximity to human dwellings (Figure 1).

As a prevention measure, we recommend avoiding contact with fruit bats and their secretions/excretions. We also encourage persons to wash and/or peel fruit, in addition to washing their hands, before preparing meals or consuming fruit. Greater understanding of the relationships between pteropid fruit bats, NiV, and its transmission to humans might offer additional strategies for safe coexistence and disease prevention for Bangladesh and other countries where fruit bats reside. Finally, because the geographic range of this highly lethal pathogen may correspond to the distribution of the genus *Pteropus*, including parts of China and Australia, most of the Indian subcontinent, and Southeast Asia (*12*,*30*), factors that promote transmission from bats to humans need to be defined and the role of person-to-person transmission needs to be better characterized.
